# Impacts of Moso bamboo (*Phyllostachys pubescens*) invasion on species diversity and aboveground biomass of secondary coniferous and broad-leaved mixed forest

**DOI:** 10.3389/fpls.2022.1001785

**Published:** 2022-09-30

**Authors:** Xi Chen, Xin Chen, Shiqi Huang, Dongming Fang

**Affiliations:** Jiyang College of Zhejiang A&F University, Zhuji, Zhejiang, China

**Keywords:** biomass, Moso bamboo invasion, *Phyllostachys pubescens*, secondary coniferous and broad-leaved mixed forest, species diversity

## Abstract

In recent decades, Moso bamboo has been largely increasing in the subtropical area of China, raising ecological concerns about its invasion into other native forest ecosystems. One concern is whether the invasion of Moso bamboo significantly simplifies forest community composition and structure and declines biomass. This study adopted the space-for-time method to investigate a secondary coniferous and broad-leaved mixed forest (SF) being invaded by an adjacent Moso bamboo forest (MB) in the Wuxie forest reserve, Zhejiang Province. Three plots were established in each SF, MB, and transitional forest. The results showed that the species composition and species dominance of the arborous layer changed significantly (*P* < 0.05), which was indicated by the significantly decreased species richness (Margalef index, Shannon–Wiener index, and Simpson index) and evenness (Pielou evenness index). In contrast, the species richness of the shrub and herbaceous layers had two divergent indications (increasing or unchanged), and the evenness remained unchanged. The total and arborous-layer aboveground biomass of the forest community has had no noticeable change (*P* < 0.05). However, the biomass of the shrub and herbaceous layers showed an increasing trend (shrub significant but herbaceous not), but they only occupied a small proportion (∼1%) of the total biomass. Finally, the aboveground biomass and the diversity index had no significant correlation in each layer and overall stands. We hope that the findings could provide a theoretical basis for the invasion mechanism and ecological consequences of the Moso bamboo invasion.

## Introduction

The invasion of alien plant species into native plant communities has become a common phenomenon worldwide over the past several decades (Ramula and Pihlaja, 2012; Wang et al., 2017, 2019). Alien plant species become the dominant species of the new communities, for instance, due to their growth advantage or lack of enemies when they invade the new ecosystem (van Kleunen et al., 2010). Alien plant species inhibit the growth of other local plants, which eventually reduces biodiversity (Davies, 2011; Vilà et al., 2011) and affects the stability of community biomass (Bai et al., 2007). Similarly, native plants may become increasingly dominant in their original range, a phenomenon known as overabundance (Garrott et al., 1993), i.e., native plant invasion. The invasion of native plants into new ecosystems in the same region or country may also significantly impact biodiversity (Ouyang et al., 2016). Compared with the extensive studies that draw attention to the impact of alien plant invasion, relatively few studies consider the effects of native plant invasion (Nackley et al., 2017; Warren et al., 2017; Shi et al., 2018).

Generally, a forest with a closed canopy can resist the invasion of other species due to the weak light conditions under the canopy, which could weaken the growth of invading seedlings. Without human intervention, native species will not encroach on adjacent natural forests. However, when shade-tolerant tree species invade, the complete forest may only provide weak resistance (Martin et al., 2009). Moso bamboo (*Phyllostachys pubescens*) is widely distributed in southern China (Shi et al., 2020) and has competitive strengths *via* its strong rhizome-root system and leaf functional traits (Huang et al., 2019, 2020; Guo et al., 2021). Native Moso bamboo may invade the surrounding natural forest by itself (Bai et al., 2016) because the elongation of bamboo shoots may depend on the carbohydrate supply of bamboo rhizomes. The reserve stored in bamboo rhizomes can hold a large amount of energy and resources for shoot growth (Xue et al., 2021), largely independent of the light environment (Wang et al., 2016).

The invasion of Moso bamboo into subtropical evergreen forests has become a significant problem in many areas of southern China (Tian et al., 2020). In the past, Moso bamboo was the essential bamboo species for both shoot and timber production in China. Additionally, the Moso bamboo forest (MB) has a comparable net primary productivity (8.86 ± 3.46 Mg C ha^–1^ year^–1^), which enables it to be a vast potential carbon pool (Lin et al., 2017). Therefore, managing pure Moso bamboo forests can bring considerable income to bamboo farmers. Thus, a large area of subtropical evergreen forest in southern China has been transformed into the MB by farmers (Chen et al., 2009). However, in recent years, due to the increase in Moso bamboo production capacity and the price decline, the management level of MB in many production areas has decreased. As a result, a large area of MB has been abandoned (Jiang et al., 2015), which has exacerbated the invasion of Moso bamboo. Previous studies have shown that Moso bamboo invasion may lead to a series of ecological and environmental problems, such as simplifying forest community composition and structure (Ouyang et al., 2016), declining species diversity (Okutomi et al., 1996; Nakai and Kisanuki, 2006; Yang et al., 2011; Akutsu et al., 2012; Bai et al., 2013; Zhang and Xue, 2018), and making the forest biomass change obviously (Yang et al., 2011; Fukushima et al., 2015; Lin et al., 2017; Song et al., 2017).

As the largest terrestrial ecosystem on earth, the forest has about half of the carbon reserves on land, which plays a vital role in maintaining the ecological balance of the whole world and human survival (Pan et al., 2011). The subtropical forest is the vegetation type mainly distributed in China, with a broad distribution area and rich biodiversity (Corlett, 2013). The relationship between species diversity and aboveground biomass of natural forest communities is still controversial (Lasky et al., 2014; Bracken et al., 2017; Silva Pedro et al., 2017). Studies have shown that species richness positively affects biomass or productivity of subtropical forests (Barrufol et al., 2013; Bruelheide et al., 2014; Cavanaugh et al., 2014) or has no effect (Wu et al., 2015). The different results may be due to the forest ecosystem’s different restoration or succession times. It may also be due to the complexity of community structure and the resource effectiveness of habitat conditions (Ali and Yan, 2017; Mori et al., 2017; Ratcliffe et al., 2017; van der Sande et al., 2017). Plant invasion can significantly change the species diversity and biomass of biological communities. However, the relationship between biodiversity changes and biomass of forest communities during the Moso bamboo invasion remains largely unknown.

Therefore, we selected a typical site where Moso bamboo has been expanding into the coniferous and broad-leaved mixed forest to investigate the community characteristics. We applied the “Space for Time” method (Pickett, 1989), which assumes that spatial and temporal variation are equivalent, so the different temporal stages of the Moso bamboo invasion were simultaneously compared in the same area. The species diversity and aboveground biomass of three adjacent forest types were investigated, i.e., (1) the native secondary coniferous and broad-leaved mixed forest (SF), (2) transition tree-Moso-bamboo forest (TF), and (3) nearly pure MB. We attempted to verify three assumptions: (1) The invasion of Moso bamboo changed the species composition and reduced the diversity of the local ecosystem; (2) Moso bamboo invasion may reduce the aboveground biomass of the forest community; (3) The changes in species diversity of forest community may have correlations with the aboveground biomass during Moso bamboo invasion. We anticipated that the findings might provide theoretical and management references to local farmers and the government.

## Materials and methods

### Study site

The study was performed at Wuxie Nature Reserve (120⋅2′40″E; 294⋅4′15″N) in Zhuji, Zhejiang Province, China. The area has a subtropical monsoon climate with a mean annual temperature of 16.3°C and an annual precipitation of 1,573 mm. The area has many soil types, mainly hilly red soil, with good soil fertility. The zonal vegetation is SF and MB. Due to the strong expansion ability of the Moso bamboo rhizome, a mixed forest of coniferous and broad-leaved trees and bamboos is formed between the two types of forests.

### Sampling design

In May 2020, we selected three transects spanning from a SF, *via* a transition zone, to a MB, representing three typical stages of the Moso bamboo invasion into subtropical forests. The altitude of the plot is 210–230 m, containing SF, representing the forest stage not invaded by Moso bamboos. The dominant species in the arborous layer of SF was *Schima superba*, with a forest age of 30–40 years. The associated species were *Liquidambar formosana* and *Pinus massoniana*. The average height of the arborous layer was 15.6 m, and the average diameter breast height (DBH) was 20.5 cm. The TF represented the forest stage moderately invaded by Moso bamboo, and the ratio of bamboo to wood is about 4:1. The average height and DBH of Moso bamboo were 16.3 m and 11.5 cm, respectively. Finally, the MB represented the forest stage heavily invaded by Moso bamboos, and the bamboos were the species with an absolute advantage in the arborous layers. The average height of Moso bamboo was 16.9 m, and the average DBH was 12.1 cm (Table 1).

**TABLE 1:** The height and diameter breast height (DBH) class of Moso bamboo in secondary coniferous and broad-leaved mixed forest (SF), transitional forest, and Moso bamboo forest (MB) (mean ± SE).

Forest type	Height (m)	DBH (cm)
Transitional forest (TF)	16.30 ± 0.16**b**	11.50 ± 0.14**b**
Moso bamboo forest (MB)	16.87 ± 0.09**a**	12.13 ± 0.10**a**

Different lowercase letters indicate significant differences at *P* < 0.05.

Each transect had a width of 30 m and a length of 80 m. The utmost 5 m surrounding each horizontal transect was set as a buffer zone, and the middle area was used for setting up three 20 m × 20 m sampling plots with a 5-m spacing between them. In each sampling plot, biomass and diversity of the arborous layer were investigated. Furthermore, two 5 m × 5 m quadrats were set at the diagonal position of each sample plot and were used for shrub layer species investigation. Additionally, one 1 m × 1 m quadrats were placed in the center, and another four were established in the four corners of the sample plot, which were used for herbaceous layer species investigation. We measured the DBH of trees (>5 cm) from the arborous layer and recorded the species, DBH, height, and abundance. Woody plants with DBH less than 5 cm were measured in the shrub layer, including saplings and shrubs, and we recorded species names, ground diameter, height, abundance, and so on.

### Aboveground biomass

The tree height, DBH, and bamboo age were measured through quadrat investigation. Then the standing volume of trees in the sample plot was estimated according to the Chinese standing volume table (Zeng, 2018), and the aboveground biomass of the arborous layer per unit area was calculated through the volume biomass model (Wang et al., 2009; Shen and Tang, 2019). The formulas are as follows:


(1) Treevolume:V=cD0Hc1+c2ε

(2) $$\mathrm{Treebiomass}:B=\frac{v}{a+b\,v}$$

(3) Bamboobiomass:W=0.0520DA2.20520.4457

where *V* is the standing volume, *D* is the diameter at breast height (DBH), *H* is the tree height, *c*_*i*_ is the parameters (*i* = 0, 1, 2), ε is the error, *B* is the tree biomass, *v* is the volume per unit area, *a* and *b* are the constants of the corresponding forest type, *W* is the bamboo stem biomass, and *A* is the bamboo age.

The aboveground biomass of the shrub and herbaceous layers was obtained using the harvest method. We randomly selected a 5 m × 5 m investigation quadrat in each sample plot as the harvest quadrat of the shrub layer. Three vegetation survey quadrats with diagonal directions in the standard plot for the herbaceous layer were selected. The aboveground shrub and herbaceous layers were harvested in the set harvesting quadrat and brought back to the laboratory. The aboveground biomass of shrubs and herbs was measured after drying at 85°C to constant weight. We calculated the aboveground biomass of the shrub/herbaceous layer per unit area of each stand.

### Species diversity

Margalef index, Shannon–Wiener index, Simpson index, and Pielou evenness index were used to estimate α diversity of community species. The formulas are as follows:


(4) Margalefindexofspeciesrichness:D=m(S-1)ln⁢N

(5) Simpsonindex:D=s∑i=1sni⁢(ni-1)N⁢(N-1)

(6) $$\mathrm{Shannon}-\mathrm{Wiener}\,\mathrm{index}:H^{\prime }=-\sum_{i=1}^{s}P\,i\,\text{ln}\,P\,i$$

(7) $$\mathrm{Pielou}\,\mathrm{evenness}\,\mathrm{index}:E=\frac{H^{\prime }}{\text{ln}\,S}$$

where *S* is the total number of species, *N* is the sum of the number of individuals of all species, *P*_*i*_ is the proportion of the number of individuals of species *i* to the total number of individuals in the community, that is, *p_*i*_* = *n*_*i*_/*N*, *n*_*i*_ is the number of individuals of species *i*.


(8) ImportancevalueIV=(relativeabundance,relativefrequency,relativesignificance)/3

### Statistical analyses

SAS 9.4 (SAS Institute Inc., Cary, NC, United States) and R Statistical Software (V4.2; R Core Team, 2022) were used to process, analyze, and graph the data.

First, the diversity indexes and biomass of the arborous layer in each sampling plot were calculated and measured. At the same time, the diversity indexes and biomass in each quadrat (5 m × 5 m for the shrub layer and 1 m × 1 m for the herbaceous layer) were calculated and measured. Then, the diversity indexes and biomass of these quadrats (two and five quadrats for the shrub and herbaceous layers, respectively) per sampling plot were averaged. In this case, we got three replicates of the diversity indexes and biomass in shrub and herbaceous layers, and they were further used for comparison between the three forest types.

Considering the limited three replicates per layer that may not meet the requirement of normal distribution by the analysis of variance (ANOVA), we selected a nonparametric test (Wilcoxon rank-sum test) to conduct the comparison in diversity indexes and biomass among SF, TF, and MB. The linear correlation between diversity index and biomass of arborous, shrub, and herb layers was separately analyzed.

Specifically, R package vegan (Dixon, 2003) was used to examine the species composition difference among the three forest types (MB, TF, and SF) with PerMANOVA (permutational multivariate ANOVA).

## Results

### Effects of Moso bamboo invasion on species composition and quantitative characteristics

The number of standing bamboo and trees in the three stands varied greatly. After the expansion of Moso bamboo, the number of standing bamboo and total standing trees in the MB increased significantly, increasing to 2,783 and 2,575 culms per hectare, respectively. The number of standing stems of other tree species decreased significantly; 208 plants/ha were reduced (Table 2). These results indicated that the number of standing bamboos increased during the expansion of Moso bamboo was significantly greater than the number of coniferous and broad-leaved mixed species decreased, resulting in a significant increase in the total number of standing individuals in the community.

**TABLE 2:** Effect of Moso bamboo invasion on stem number [ind./ha; (*N* = 3 in each group)].

Expansion phase	Forest type	Stems of Moso bamboo	Stems of other trees	Total stems
Early stage	SF	0.0 ± 0.0	483.3 ± 28.9	483.3 ± 28.9
Middle stage	TF	1,833.3 ± 624.7	441.7 ± 118.1	2,275.0 ± 633.9
Later stage	MB	2,783.3 ± 797.4	275.0 ± 75.0	3,058.3 ± 821.7
Changes between early and late stages		2,783.3 ± 797.4	−208.3 ± 101.0	2,575.0 ± 844.5

**FIGURE 1:**
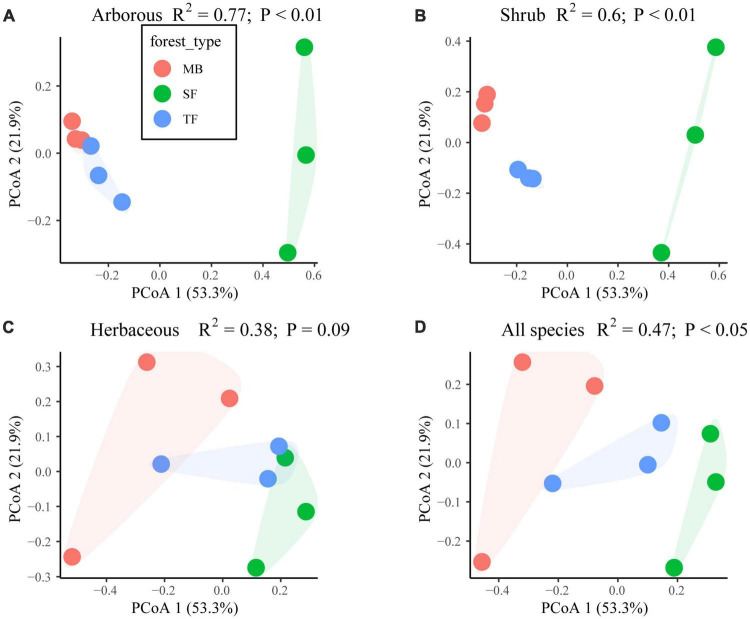
Effect of the Moso bamboo invasion on species composition difference of each layer among the three forest types [Moso bamboo forest (MB), transition tree-Moso-bamboo forest (TF), and secondary coniferous and broad-leaved mixed forest (SF)] in **(A)** arborous, **(B)** shrub, **(C)** herbaceous layers, and **(D)** the compound layers with all arborous, shrub, herbaceous species. Significant differences examined with PerMANOVA (permutational multivariate ANOVA) are indicated by *P* < 0.05 or 0.01. PCoA means principal co-ordinates analysis. PCoA1 and PCoA2 in the figures represented two components of PCoA with largest explaining power to the variation of the species composition difference.

Except in the herbaceous layer (*P* > 0.05), the species composition in the arborous, shrub, and whole layers was significantly different among the three communities (*P* < 0.05; Figure 1 and Table 3).

**TABLE 3:** Effects of Moso bamboo invasion on main species composition and importance value (mean ± SD).

Layer	Forest type	Species	Important value
Arborous	SF	*Schima superba*	27.6 **±** 15.3
		*Liquidambar formosana*	27.1 **±** 11.4
		*Pinus massoniana*	12.5 **±** 5.7
		*Castanopsis eyrei*	6.2 **±** 2.6
		*Trachycarpus fortunei*	6.1 **±** 7.4
		*Castanopsis sclerophylla*	5.7 **±** 3.6
		*Ligustrum lucidum*	3.4 **±** 2.7
		*Cunninghamia lanceolata*	3.4 **±** 2.7
		*Cyclobalanopsis glauca*	2.7 **±** 1.5
		*Liriodendron chinense*	2.7 **±** 1.5
		*Michelia figo*	2.6 **±** 1.3
	TF	*Phyllostachys pubescens*	48.2 **±** 7.8
		*Pinus massoniana*	15.6 **±** 7.2
		*Schima superba*	12.7 **±** 2.0
		*Cyclobalanopsis glauca*	6.2 **±** 0.7
		*Cunninghamia lanceolata*	3.9 **±** 0.7
		*Castanopsis sclerophylla*	3.6 **±** 0.4
		*Liquidambar formosana*	2.4 **±** 1.5
		*Trachycarpus fortunei*	1.9 **±** 0.6
		*Myrica rubra*	1.8 **±** 0.4
		*Elaeocarpus decipiens*	1.8 **±** 0.4
		*Ligustrum lucidum*	1.7 **±** 0.3
	MB	*Phyllostachys pubescens*	64.9 **±** 2.9
		*Schima superba*	14.1 **±** 0.1
		*Pinus massoniana*	9.3 **±** 0.3
		*Cyclobalanopsis glauca*	8.6 **±** 2.7
		*Cunninghamia lanceolata*	3.1 **±** 0.6
Shrub	SF	*Indocalamus tessellatus*	31.6 **±** 30.8
		*Ligustrum lucidum*	7.4 **±** 3.9
		*Quercus glauca*	7.0 **±** 4.3
		*Lindera glauca*	5.2 **±** 4.4
		*Symplocos stellaris*	4.5 **±** 2.4
	TF	*Camellia japonica*	11.6 **±** 3.5
		*Symplocos sumuntia*	8.9 **±** 2.7
		*Cinnamomum japonicum*	7.0 **±** 4.8
		*Symplocos stellaris*	6.9 **±** 6.3
		*Ligustrum lucidum*	5.8 **±** 1.2
		*Quercus glauca*	5.7 **±** 2.2
	MB	*Symplocos stellaris*	12.3 **±** 3.9
		*Camellia japonica*	9.3 **±** 4.3
		*Symplocos sumuntia*	9.2 **±** 1.6
		*Ilex chinensis*	6.5 **±** 5.4
		*Lithocarpus glaber*	5.7 **±** 6.5
Herbaceous	SF	*Rubus buergeri*	70.8 **±** 6.1
		*Ophiopogon bodinieri*	10.2 **±** 9.4
		*Nephrolepis auriculata*	7.2 **±** 4.3
		*Lepidomicrosorum buergerianum*	6.1 **±** 2.3
		*Anredera cordifolia*	5.8 **±** 1.7
	TF	*Rubus buergeri*	57.5 **±** 11.6
		*Lepidomicrosorum buergerianum*	16.3 **±** 0.7
		*Nephrolepis auriculata*	15.3 **±** 12.0
		*Ophiopogon bodinieri*	6.6 **±** 5.7
		*Plantago depressa*	4.3 **±** 1.8
	MB	*Rubus buergeri*	45.3 **±** 13.4
		*Lepidomicrosorum buergerianum*	15.1 **±** 8.0
		*Pyrrosia lingua*	12.7 **±** 6.9
		*Nephrolepis auriculata*	9.3 **±** 10.8
		*Tetrastigma formosanum*	7.1 **±** 7.1
		*Gardneria multiflora*	5.4 **±** 4.1
		*Anredera cordifolia*	5.1 **±** 3.6

In red, top-contributing species; in blue, species descending from the top-contributing nodes.

For the arborous layer, species richness decreased from 11 in SF and TF to 5 in MB. Only three species stayed in all three communities, i.e., *S. superba*, *P. massoniana*, and *Cyclobalanopsis glauca*, which corresponded to dominant, inferior, and minority roles in SF with important values of 27.6, 12.5, and 2.7, respectively (Table 3). The dominant species changed from *S. superba* and *L. formosana* in SF (important values of 27.6 and 27.1, respectively) to Moso bamboo in TF and MB (important values of 48.2 and 64.9, respectively). The top contributing species *S. superba* in SF descended to an inferior role in TF and MB (important values of 12.7 and 14.1, respectively), and *L. formosana* descended to an ignorable status in TF (important value of 2.4) and disappeared in MB. *P. massoniana* stayed relatively stable in all three communities, with important values of 12.5, 15.6, and 9.3 in SF, TF, and MB, respectively. *C. glauca* is always kept as a minority, although its important values increased over the invasion stages (2.7, 6.2, and 8.6 in SF, TF, and MB, respectively).

The shrub layer’s species richness was relatively stable (5–6 species) over three communities, but none of them grew in all three communities (Table 3). In addition, the species composition differed significantly among the three forest types (*P* < 0.05; Figure 1 and Table 3). The dominant species were *Indocalamus tessellatus*, *Camellia japonica*, and *Symplocos stellaris* in SF, TF, and MB, respectively (important values of 31.6, 11.6, and 12.3, respectively). Compared with SF, the species composition was more even in both TF (important values ranging from 5.7 to 11.6) and MB (important values ranging from 5.7 to 12.3).

In the herbaceous layer of the three communities, the species richness was close to each other (five in SF and TF, and seven in MB), and the three communities had three of the same species (*Rubus buergeri*, *Nephrolepis auriculata*, and *Lepidomicrosorum buergerianum*). The statistical results indicated similar species composition among the communities (*P* > 0.05; Figure 1 and Table 3). Furthermore, the dominant species were the same, i.e., *R. buergeri*, but its important values dropped with the invasion progression (70.8, 57.5, and 45.3 in SF, TF, and MB, respectively).

### Effects of the Moso bamboo invasion on species diversity

#### Changes in species diversity in the arborous layer

**FIGURE 2:**
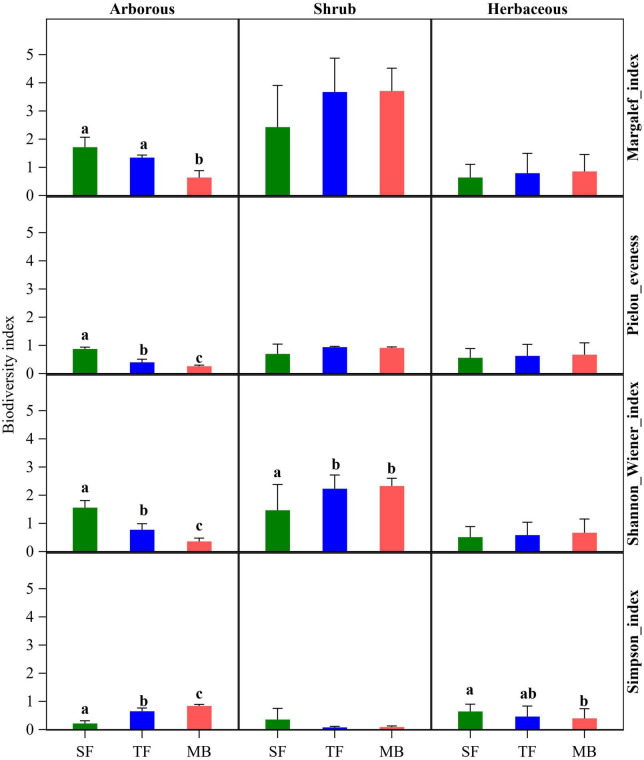
Effect of the Moso bamboo invasion on species diversity of each layer. Different letters in the same column indicate significant differences at *P* < 0.05.

During the invasion of Moso bamboo into SF, species diversity in the arborous layer showed a significant decreasing trend, indicated by the significantly decreasing Shannon–Wiener index and increasing Simpson index over SF, TF, and MB (*P* < 0.05; Figure 2). Changes in the Margalef index seemed to support the same trend, but its values were not significantly different between the SF and TF (*P* > 0.05; Figure 2). Moreover, as the invasion progressed, the species’ evenness got worse, as indicated by the reducing Pielou index (*P* < 0.05; Figure 2).

#### Changes in species diversity in the shrub layer

The Margalef index and Simpson index suggested no significant difference in the shrub layers among the three communities (*P* > 0.05; Figure 2), although the values seemed to point to increasing biodiversity over the invasion. In contrast, the Shannon–Wiener index indicated significantly higher species diversity in TF and MB than in SF (*P* < 0.05; Figure 2). The species evenness had no significant difference in the shrub layers among the three communities, as indicated by the Pielou index (*P* > 0.05; Figure 2).

#### Changes in species diversity in the herbaceous layer

The Margalef and Shannon–Wiener indexes suggested no difference in the herbaceous layers among the three communities (*P* > 0.05), while the Simpson index indicated higher diversity in MB than in SF (*P* < 0.05; Figure 2). Finally, species evenness had no significant difference in the herbaceous layers among the three communities, as indicated by the Pielou index (*P* > 0.05; Figure 2).

### Effects of Moso bamboo invasion on aboveground biomass

During the invasion of Moso bamboo into SF, the aboveground biomass showed no significant differences in arborous and herbaceous layers and the overall stand between the three forest types (*P* > 0.05; Table 4). In contrast, the aboveground biomass in the shrub layers was significantly higher in TF and MB than in SF (*P* < 0.05; Table 4). The proportions of aboveground biomass in each layer to the total biomass had no significant difference among the three forest types (*P* > 0.05; Table 4). The arborous layers occupied the largest proportion (∼99%) of the aboveground biomass in all three forest types. With the invasion progress, the aboveground biomass of Moso bamboo in the community gradually increased to 3,538.63 ± 1,305.82 and 7,482.72 ± 2,532.43 g m^–2^ in the TF and MB, which correspondingly occupied 17.90 ± 6.41% and 41.56 ± 8.21% of the total aboveground biomass, respectively.

**TABLE 4:** Effect of the Moso bamboo invasion on aboveground biomass (mean ± SD; *N* = 3 in each group) in each layer and their contribution to the total aboveground biomass.

	Forest type	Total aboveground biomass	Arborous (trees and Moso bamboo)	Shrub	Herbaceous	Moso bamboo
Biomass (**g m^–2^**)	SF	19,529.92 ± 6,190.68	19,393.52 ± 6135.53	16.72 ± 1.52**a**	119.68 ± 57.48	–
	TF	19,679.57 ± 675.28	19,533.55 ± 683.46	22.37 ± 8.95**b**	123.64 ± 30.91	3,538.63 ± 1,305.82
	MB	17,749.90 ± 4158.71	17,557.27 ± 4,112.54	56.21 ± 27.78**b**	136.42 ± 24.22	7,482.72 ± 2,532.43
percent (%)	SF		99.31 ± 0.09%	0.09 ± 0.03%	0.59 ± 0.12%	–
	TF		99.26 ± 0.13%	0.11 ± 0.05%	0.63 ± 0.16%	17.90 ± 6.41%
	MB		98.91 ± 0.13%	0.31 ± 0.10%	0.78 ± 0.07%	41.56 ± 8.21%

Different letters in the shrub column indicate significant differences among the forest types in the same layer at *P* < 0.05.

### Relationship between diversity and aboveground biomass

The correlation between plant diversity and aboveground biomass was insignificant in the arborous, shrub, and herbaceous layers (*P* > 0.05; Table 5). It was also not significant in the overall stand when all layers were considered (*P* > 0.05; Table 5).

**TABLE 5:** Effect of the Moso bamboo invasion on the relationships between aboveground biomass and biodiversity indexes in each layer and the whole stand comprised of three layers (*N* = 9 in each group).

Forest type	Biodiversity indexes	*Pr > F*	*R* ^2^
Arborous	Margalef_index	0.81	0.01
	Pielou_eveness	0.90	0.00
	Shannon_Wiener_index	0.97	0.00
	Simpson_index	0.97	0.00
Shrub	Margalef_index	0.30	0.15
	Pielou_eveness	0.59	0.04
	Shannon_Wiener_index	0.31	0.15
	Simpson_index	0.50	0.07
Herbaceous	Margalef_index	0.22	0.21
	Pielou_eveness	0.08	0.38
	Shannon_Wiener_index	0.20	0.22
	Simpson_index	0.22	0.21
All species	Margalef_index	0.34	0.13
	Pielou_evenness_index	0.09	0.36
	Shannon_Wiener_index	0.22	0.21
	Simpson_index	0.14	0.28

## Discussion

### Effects of Moso bamboo invasion on species composition and diversity

Species composition is not only the basis of community structure and biodiversity but also the root of forest ecosystem processes (Tilman et al., 1997). Existing studies show that both alien and native plant invasions have a certain impact on the community’s species composition (Garrott et al., 1993; Davies, 2011; Lima et al., 2012). This study found that the invasion of Moso bamboo significantly changed the species composition in the arborous and shrub layers but not in the herbaceous layer (Figures 1A–C and Table 3), which also changed the overall species composition significantly (Figure 1D). For example, the number of species had been reduced from 11 in SF to 5 in MB in the arborous layer. The important values of the main dominant arborous species *S. superba*, *L. formosana*, and *P. massoniana* decreased from 27.6, 27.1, and 12.5 before expansion to 14.1, 0, and 9.3, respectively (Table 3). On the contrary, Moso bamboo gradually dominated the arborous layers with important values of 48.2 and 64.9 in TF and MB, respectively. The interchanged dominance between trees and Moso bamboo in the arborous layer was consistent with several other research results (Bai et al., 2013; Yang et al., 2015; Ouyang et al., 2016). The findings supported our first hypothesis, i.e., that the invasion of Moso bamboo changed species composition, especially in the arborous and shrub layers. Although a few herbaceous species disappeared or newly appeared with the invasion, the species composition in the herbaceous layer was not significantly changed (Figure 1D and Table 3). Three herbaceous species (*R. buergeri*, *N. auriculata*, and *L. buergerianum*) have always been in the SF, TF, and MB, and *R. buergeri* has always been the most dominant species in the herbaceous layers of the three communities. The finding may indicate that the invasion of Moso bamboo did not alter the near-ground light or soil conditions suiting the local shade-tolerant herbaceous species.

Regarding species diversity of the three layers, the arborous layer was the most influenced by the invasion in this study. Compared with the pre-invaded SF, MB had significantly lower diversity, as indicated by all three diversity indexes (Margalef, Shannon–Wiener, and Simpson index; *P* < 0.05; Figure 2). At the same time, the species evenness was smaller in MB than in SF (*P* < 0.05; Figure 2). This result is consistent with several previous studies (Bai et al., 2013; Yang et al., 2015; Ouyang et al., 2016) and partly supports our first hypothesis that invasion decreases plant diversity. However, the first hypothesis was not fully supported by the results regarding the shrub and herbaceous layers. Among the three diversity indexes, only one of them (Shannon–Wiener and Simpson indexes for the shrub and herbaceous layers, respectively) indicated a significantly increased diversity. In contrast, the other two indexes inversely meant that the diversities of these two layers were unchanged (Figure 2). The finding was consistent with the results observed in the invasion progress of Moso bamboo into Chinese fir (Xu et al., 2019). In contrast, compared with the invaded coniferous and broad-leaved mixed forests (Bai et al., 2013; Yang et al., 2015; Ouyang et al., 2016) and the secondary evergreen broad-leaved forest (Ouyang et al., 2016), MB had lower diversity in both shrub and herbaceous layers. In another study on the invaded coniferous and broad-leaved mixed forests (Bai et al., 2013), species diversity was reduced in the shrub layers but improved in the herbaceous layer.

Species diversity varied in different studies, which may be related to the short observation time and the different invasion stages because understory species diversity is a dynamic process. The increase of species diversity in shrub and herb layers during the invasion of Moso bamboo into SF may result from increased light intensity under the forest (Liu et al., 2011) because better light conditions may reduce light competition to improve the species diversity of understory vegetation (Hautier et al., 2009). However, it should be noted that different plants have different adaptability to light, and the influence of light intensity on species diversity may be restricted by the light requirement of plants (Boothroyd-Roberts et al., 2013). Much higher or much lower light intensity is not conducive to increasing the species diversity of understory plants (Tao and Feng, 1998). On the contrary, Moso bamboo forests have low stem density and soil-water content than other forest types (Shinohara and Otsuki, 2015), which may benefit understory diversity. In addition, Moso bamboo invasion increased soil bacterial diversity, soil pH, and soil organic carbon concentrations (Lin et al., 2014; Liu et al., 2021; Ouyang et al., 2022), which may also be beneficial to increasing the biodiversity under the forest. However, there is not enough direct evidence to support this argument. The mechanism of Moso bamboo invasion needs further study.

### Effects of Moso bamboo invasion on aboveground biomass and its relationship with species diversity

Although the response relationship between the change of community species composition and forest ecosystem function is complex, many studies have shown that the loss of community species will lead to the decline of forest productivity and resistance and increase the instability of the system (Lavorel and Garnier, 2002; Barrufol et al., 2013; Bruelheide et al., 2014; Cavanaugh et al., 2014). In particular, the replacement of the dominant species in a community will affect the community environment on which other tree species depend, leading to the transformation of plant functional groups (Hooper et al., 2005), thus affecting the composition, structure, and function of the whole ecosystem (Lima et al., 2012). Contrary to our second hypothesis, the aboveground biomass in the entire stand and the arborous layer was not significantly reduced in TF and MB compared to the SF, which may be attributed to the increasing stem number of Moso bamboo (Table 2). This finding is consistent with the research results of Fukushima et al. (2015).

Previous studies have shown that Moso bamboo may obtain more nutrients and light resources by increasing the DBH and height to promote rapid growth and expansion during the invasion (Bai et al., 2012; Liu et al., 2016; Xu et al., 2019). However, in this study, the average DBH and height of Moso bamboo decreased during the invasion of Moso bamboo into SF (Table 1). This result may be due to the different invasion sites and forests (Liu et al., 2019). It may also be that the intraspecific competition of standing bamboo in the MB may be more intense, so it is necessary to obtain more light and nutrients by increasing height and DBH to get a competitive advantage. Its internal mechanism needs to be further studied.

The proportion of understory vegetation biomass in the total biomass of the forest ecosystem is small. Still, it plays a vital role in maintaining the succession and functional stability of the whole forest ecosystem (Sun et al., 2015). This study found that the aboveground biomass of the shrub and herbaceous layers and their proportion of the total aboveground biomass increased during the invasion of Moso bamboo to SF, but the increment in the herbaceous layer was not statistically significant (Table 4). Although the shrub and herb layers of the three stands contribute less to the biomass of the community, the understory often has more abundant species (Table 3). These species play an important role in the environment under plastic afforestation, affecting forest dynamics, soil carbon pool, and nutrient cycle (Nilsson and Wardle, 2005). Therefore, the change of species biomass in the understory should not be ignored when evaluating the ecological aftereffects of the Moso bamboo invasion.

The results showed no significant correlation between aboveground biomass and the diversity index of each layer and the whole stand during the Moso bamboo invasion (*P* > 0.05; Table 5), which indicated that the Moso bamboo invasion had no impact on the relationship. This result does not verify our third hypothesis that the positive correlation between species diversity and biomass is common across forest layers (Zhang et al., 2017). This insignificant relationship may be attributed to the intensity and direction of the correlation between biodiversity and ecosystem function changing under different environmental conditions (Cardinale et al., 2000). According to the stress gradient hypothesis, species are more competitive in the ideal environment but more mutual aid in an adverse environment. Therefore, it is more likely to find a positive correlation between species richness and aboveground biomass in adverse environmental conditions (Maestre et al., 2009). In this study, the arborous layer is rich in light resources. Therefore, Moso bamboo contributes a lot of biomass and excludes the survival of other species, which leads to no significant positive relationship between species richness and biomass.

## Conclusion

Although the Moso bamboo invasion has drawn considerable attention to its impact, especially on biodiversity, few studies have reported the effects of the Moso bamboo invasion on forest biomass and the forest biodiversity–biomass relationship. By using the space-for-time method, we investigated the dynamics of species composition, diversity, and aboveground biomass of arborous, shrub, and herbaceous layers during Moso bamboo invasion into a SF. As the most significant part of the aboveground biomass (>99%), the arborous layer showed a decreasing trend in the aboveground biomass with the invasion progress. Still, the decrease was not statistically significant, which may be attributed to the increased five-fold stems. Moreover, the aboveground biomass in the arborous layer had no significant correlation with the significantly decreasing diversity. In the understory layers, shrubs had significantly increased aboveground biomass, while herbs did not. Similar to the arborous layer, both shrub and herbaceous layers did not significantly correlate their aboveground biomass and diversity indexes. However, these findings were concluded based on three plots per forest type and one transitional status, which may not cover enough variation in both biodiversity and aboveground biomass. Additionally, belowground biomass is also an essential part of the forest, which should be considered. We hope the findings of this study contribute to some fundamentals for future research and understanding of Moso bamboo invasion.

## Data availability statement

The original contributions presented in this study are included in the article/supplementary material, further inquiries can be directed to the corresponding author/s.

## Author contributions

XiC: methodology, software, investigation, visualization, writing – original draft and review, and editing. XinC and SH: data curation and investigation. DF: methodology, writing – review and editing, conceptualization, and investigation. All authors contributed to the article and approved the submitted version.
